# A Case of Hæmaturia

**Published:** 1907-09-28

**Authors:** G. Ainsworth


### The General Practitioner's Column.

[Contributions to this Column are invited, and if accepted will be paid for.]

### A CASE OF HÆMATURIA.

By G. AINSWORTH, M.B., Ch.B.

The following case is interesting from the doubt as to the correct diagnosis, from its long duration, and from its resistance to treatment.

The patient, T. R., is a boy 13 years of age: from both parents he has a rheumatic family history. Four months ago he was taken ill with an "influenzal cold," which, however, did not prevent him from working. A few days later his throat became sore and swallowing was painful. The tonsils and mouth were inflamed and swollen, the cervical glands enlarged, and he suffered from headache, nausea, and slight pyrexia. These symptoms were succeeded by swelling of the ankles and wrists, aching of the limbs, and erythematous patches on the legs. The joints were affected in succession, and were hot and tender; at the same time the temperature rose to 102°-103°, and continued so for a day or two. Under the influence of rest in bed and moderate doses of salicylate of soda the symptoms subsided, the throat recovered, and the glandular swellings diminished.

### The Onset of Urinary Symptoms.

With convalescence the urine became thick and greenish in colour, small in quantity, and of a high specific gravity. In a day or two the colour changed to red; the reaction was acid and bright blood without clots and was uniformly mixed with the urine. Microscopically were seen numerous red corpuscles, including megalocytes, poikilocytes, and nucleated cells; also epithelial and a few granular casts. Micturition was frequent, though but little was voided each time; the act was not attended by pain: palpation of the kidneys revealed no enlargement or tenderness, nor was there any lumbar aching. About the time of the onset of hæmaturia, epistaxis, sickness and giddiness were noted: there was no headache and no pyrexia, and sight remained good. Hæmaturia continued for some weeks, during which time the patient became more anæmic. There was occasional pain and palpitation in the cardiac region, and about one month after the commencement of the illness a systolic bruit was noticed, well marked and localised to the apex, which was in the fifth costal interspace in the nipple line; there was no reduplication of the second sound.

### The Treatment.

The patient was kept warm in bed and lived chiefly on milk and barley water; his bowels were very costive, and the hæmaturia, which seemed to be worse when there was constipation, was somewhat relieved after purgation. As regards medicinal treatment, liquid extract of ergot with digitalis was first given; the dose of the former was gradually increased from 5 to 10 minims. In a few days tincture of hamamelis in 10 drop doses was added, but no improvement resulted. Tannic and gallic acids were next tried, but were discontinued, as nausea and constipation became worse. Adrenalin solution in 5 minim doses, afterwards increased to 10 minims, seemed to cause a very temporary improvement, followed by relapse and pain in the loins. Lastly, chloride of calcium was ordered in 30 grain doses every two hours: as improvement followed, this frequency was steadily diminished. In two weeks the urine was practically normal, the heart improved, anæmia subsided, and the patient was allowed up two months after the murmur first appeared.

### What was the Case?

The question of diagnosis is still not quite clear. The initial disorders were almost certainly manifestations of acute rheumatism; there was never any ground for suspicion of scarlet fever or diphtheria, nor was the condition of the throat suggestive of acute streptococcal invasion. If the explanation of the cardiac murmur be the occurrence of rheumatic endocarditis, a renal infarction would easily account for urinary symptoms; but against this there was no sudden pain in the loin, nor was the hæmaturia absolutely abrupt in onset and in resolution. Acute nephritis, on the other hand, is not a common complication of a subsided articular rheumatism, and would almost certainly have been attended with considerable dropsy and headache. Passive congestion of cardiac origin is scarcely consistent with the condition of the heart, which at no time exhibited signs of failure. Again, hæmaturia sufficient to cause marked anæmia suggests the possibility of several emboli having lodged in the renal arteries; if that were so, one would expect to have evidence of a similar event in other organs. Whether recovery was assisted by, or merely coincident with, the administration of calcium chloride is another rather difficult point in an interesting case.

